# Use of Ordered Beta Regression Unveils Cognitive Flexibility Index and Longitudinal Cognitive Training Signatures in Normal and Alzheimer’s Disease Pathological Aging

**DOI:** 10.3390/brainsci14050501

**Published:** 2024-05-15

**Authors:** Daniel Alveal-Mellado, Lydia Giménez-Llort

**Affiliations:** 1 Institut de Neurociències, Universitat Autònoma de Barcelona, 08193 Cerdanyola del Vallès, Barcelona, Spain; daniel.alveal@autonoma.cat; 2 Department of Psychiatry and Forensic Medicine, School of Medicine, Universitat Autònoma de Barcelona, 08193 Cerdanyola del Vallès, Barcelona, Spain

**Keywords:** behavioral studies, data analysis, Alzheimer’s disease, aging, Morris Water Maze, search strategies, cognitive flexibility

## Abstract

Generalized linear mixed models (GLMMs) are a cornerstone data analysis strategy in behavioral research because of their robustness in handling non-normally distributed variables. Recently, their integration with ordered beta regression (OBR), a novel statistical tool for managing percentage data, has opened new avenues for analyzing continuous response data. Here, we applied this combined approach to investigate nuanced differences between the 3xTg-AD model of Alzheimer’s disease (AD) and their C57BL/6 non-transgenic (NTg) counterparts with normal aging in a 5-day Morris Water Maze (MWM) test protocol. Our longitudinal study included 22 3xTg-AD mice and 15 NTg mice (both male and female) assessed at 12 and 16 months of age. By identifying and analyzing multiple swimming strategies during three different paradigms (cue, place task, and removal), we uncovered genotypic differences in all paradigms. Thus, the NTg group exhibited a higher percentage of direct search behaviors, while an association between circling episodes and 3xTg-AD animals was found. Furthermore, we also propose a novel metric—the “Cognitive Flexibility Index”—which proved sensitive in detecting sex-related differences. Overall, our integrated GLMMs-OBR approach provides a comprehensive insight into mouse behavior in the MWM test, shedding light on the effects of aging and AD pathology.

## 1. Introduction

Experimental research commonly requires sophisticated statistical methodologies capable of handling non-normally distributed data collected from dependently sampled observations, such as those clustered into groups or with repeated observations from the same individuals (i.e., longitudinal studies) [1,2]. As a result, there has been an increase in the use of innovative data analysis strategies including generalized linear mixed models (GLMMs). This methodology allows for the analysis of response variables from different distributions in longitudinal studies by including so-called random effects [3,4,5,6,7]. Although GLMMs have been used mainly to treat binary and categorical data [8], they have been recently applied in ecological and behavioral studies that gathered continuous response data such as percentages or ratios [9,10,11]. Previously, the beta regression model was proposed as an accurate method for handling percentage data [12]. Nevertheless, the model struggles with calculating degenerated data, wherein the range of values includes 0 or 1. In response, Kubinec [13] introduced the ordered beta regression model, which is capable of handling degenerated data. Nowadays, the continuous advancements in statistical software, such as R Software, offers the potential to amalgamate GLMMs and ordered beta regression models [13,14]. In our laboratory, we consider that this novel approach has broad applications, including handling various tests for assessing animal behavior, such as the neuroethological ones used in our translational behavioral neuroscience research. Notably, we hypothesize that applying this methodology to the Morris Water Maze (MWM) test, a common method for evaluating spatial learning and memory in rodents, might be highly beneficial in solving the complexity of data analysis of search strategies.

Generally, the MWM test is used to evaluate spatial cognitive performance in rodents and measure positive or negative effects of pharmacological and non-pharmacological preventive and therapeutic interventions in cognitive function; it can also be used to track the progression of cognitive dysfunction in models of neurological and psychiatric diseases. Thus, test performance enhancements are considered to be an indicator of therapeutic efficacy [15,16]. Despite several reported test protocols in the literature [17,18,19], a standard procedure typically involves three stages. The first stage, known as the visual perceptual learning or CUE stage, requires mice to swim in an opaque water-filled circular pool to reach a visible platform. The subsequent place task (PT) stage submerges and relocates the platform, requiring animals to search for it using external references. In the final probe trial or removal (RM) stage, the platform is removed, and the time animals spend in the previous platform location is analyzed.

Classical parameters such as escape latency, distance traveled, and mean speed are typically used to interpret an animal’s performance and infer its cognitive state in these paradigms [20]. However, criticisms have been raised about relying solely on these parameters as they may not provide meaningful information on animal behavior [21,22,23]. Therefore, researchers have proposed incorporating the analysis of swimming strategies adopted by animals into the standard MWM test protocol [24,25]. This approach aims to reveal behavioral disparities and memory deficits, enhancing our understanding of subtle signs of cognitive impairments. Our studies [26,27] and several others [28,29,30] showed that animals adopt a variety of swimming strategies during the test. These include search (i.e., thigmotaxis, random search, scanning, chaining, focal search, focal wrong, perseverance, and direct search) and non-search strategies (i.e., circling and floating).

Non-search strategies were previously highlighted as atypical behaviors in the test [31,32,33], indicating reduced attention to the platform search. Therefore, these are seen as confounding elements in the test analysis [34]. In addition, a correlation has been established between search strategies and different learning stages of animals [26,27,30]. This suggests that the spatial learning process evolves from an initial stage of self-centered or “egocentric” navigation, relying on sensorimotor information, to a later stage of “allocentric” navigation. In the latter stage, the animals’ trajectory is based on a non-self-centered cognitive map, where the hippocampus plays a significant role [30,35].

Cognitive flexibility, another potential aspect to evaluate in the test, involves the adaptation of behavior to new circumstances based on prior knowledge [36]. This concept has been proposed as a relevant measure to consider during the PT stage where animals are required to learn a new platform location [37,38]. Then, the inclusion of a swimming strategies analysis provides an opportunity to further investigate cognitive flexibility by examining the transition among different strategies.

The effect of prior experience in the test is another factor to consider when undertaking animal model assessment. A retest can impact performance, fluctuating based on mouse strain characteristics [39]. Longitudinal studies involving models of Alzheimer’s Disease (AD) [40,41] observed remarkable test performance stability, suggesting a potential training influence from the repeated test battery administration.

Based on our own experience in animal behavior analysis, we acknowledge the difficulty in differentiating AD and age-related impairments in mice in the MWM test. Challenges are due to the complex interplay of age, sex, and behavioral variability. Previously, our research suggested that the inclusion of multiple strategies within a single trial in the test may be an effective method to identify behavioral disparities in mice models of accelerated aging and in the triple-transgenic (3xTg-AD) model [26,27]. The 3xTg-AD model was created at the University of California, Irvine [42], and has been shown to have high face and construct validity [43,44].

Recent studies have expanded the multi-strategy approach by incorporating the classification of swimming strategies in other AD-like models [45,46]. Despite these advancements, the field lacks statistical methods to uncover the factors influencing these strategies and their evolution in longitudinal designs. Therefore, the aim of this study is to explore whether the application of GLMMs to a multi-strategy approach can identify differences by sex, genotype, or age in a group of normal (gold standard wild-type C57BL/6strain) and AD-pathologically aged mice (3xTg-AD mice) that may have been overlooked in traditional variable analysis.

## 2. Materials and Methods

### 2.1. Animals

Fifty-nine mice were used in the longitudinal experimental design. Twenty-two died during the follow-up period from 12 to 16 months of age, and finally, 37 were considered in the pre–post analysis including 22 3xTg-AD animals (*n* = 14 males and *n* = 8 females) from the Spanish colony established at the Universitat Autònoma de Barcelona, Barcelona, Spain, in a C57BL/6 background strain [47] and their 15 non-transgenic (NTg) counterparts (*n* = 8 males and *n* = 7 females). All groups were assessed at 12 and 16 months of age.

All animals were kept under standard laboratory conditions in macrolon cages (35 cm × 35 cm × 25 cm) with ad libitum food and water, at a temperature of 22 ± 2 °C and 50–70% humidity on a 12/12 h light/dark cycle starting at 8 a.m.

### 2.2. Morris Water Maze Protocol

The MWM test protocol was adapted from a previous study [48] and consisted of one day of the CUE stage, four consecutive days of the PT stage, and an RM stage on day 5.

Day 1 (CUE stage): Animals were trained to locate a visible platform (1 cm above the opaque water surface) situated in the northeast (NE) quadrant, which was marked with a black striped flag. External cues were not provided, and each animal completed four trials. 

Days 2–5 (PT1-PT4 stage): The platform was submerged 1 cm below the water level and repositioned in the opposite quadrant (southwest, SW). External cues were present, and four consecutive trials were conducted daily. Mice were introduced into the pool from different starting points (north, south, west, and east) for each trial.

Day 5 (RM stage): The platform was removed 2.5 h after the last trial on PT4, and a single trial was performed.

Throughout the experiment, mice were gently placed into the pool facing the wall and allowed to swim for a maximum of 60 s per trial. When animals failed to find the platform in 60 s, they were gently guided to it and remained standing there for 10 s.

#### 2.2.1. Classical MWM Test Analysis

Classic measurements (namely, escape latency, distance traveled, and mean speed) were automatically calculated at all stages using the video tracking software ANY-MAZE version 6.33. Additionally, during the RM stage, the time spent in each pool quadrant and in a zone surrounding 1.5 cm of the previous platform location was recorded.

#### 2.2.2. Swimming Strategy Classification

**Figure 1:**
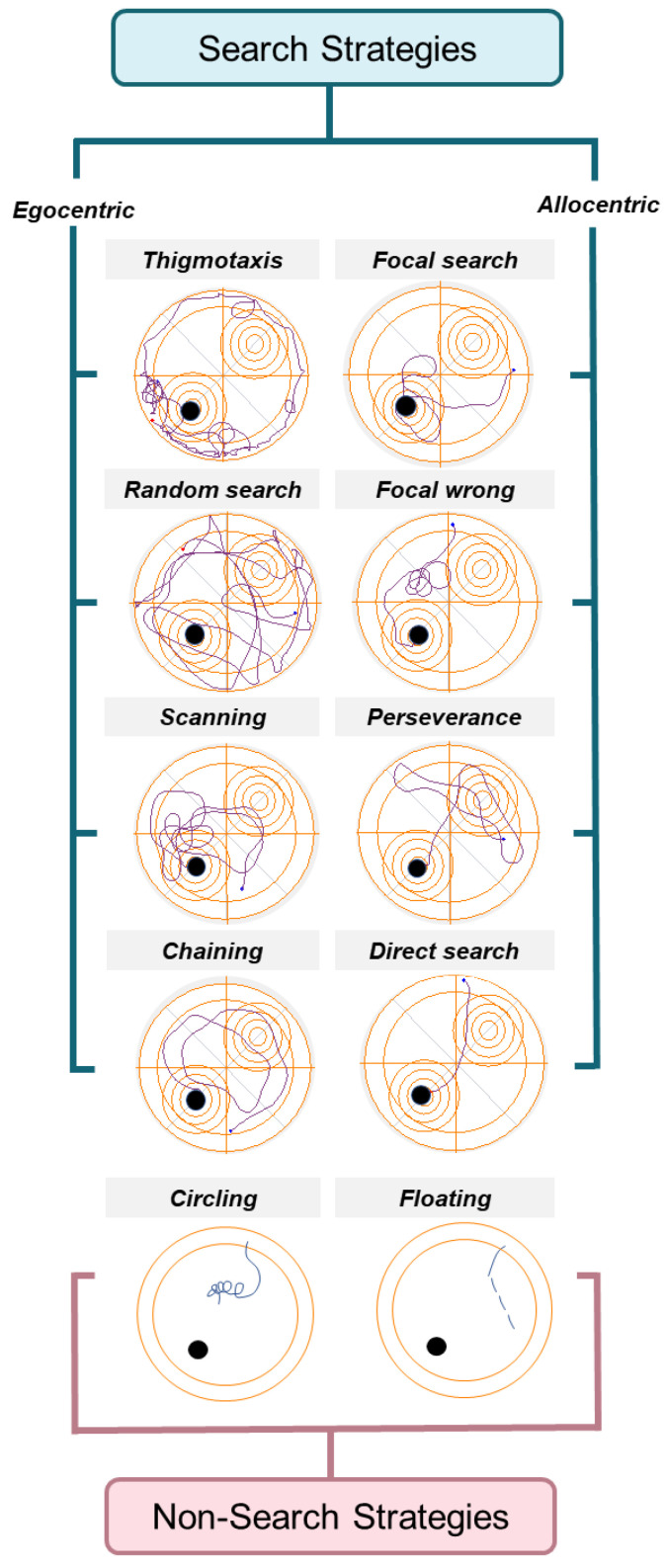
Swimming strategy classification. The swimming strategies performed by the animals in the MWM test were categorized into search and non-search strategies. Search strategies, based on hippocampal involvement, were further divided into two types including egocentric dominance and allocentric dominance.

Swimming patterns were visually identified based on the track plots recorded by ANY-MAZE. Patter identification was undertaken by an observer trained for such a task and blind to the animal’s age, sex, and genotype. The initial and final trials (T1 and T4) were considered for the analysis since they represent the main changes in the animals at each stage [48]. Swimming strategies were classified according to previous reports [30,48] (see Figure 1):

Non-search Behavior:

(a) Circling: Animals turn around their own axis in short loops without clear directionality.
(b) Floating: The animal remains inactive, not swimming in a forward motion.

Search Behavior:

Search behavior was further divided into eight distinct swimming strategies as follows:

(a) Thigmotaxis: A swim pattern performed near the pool walls or within an external ring accounting for 10% of the pool surface.
(b) Random search: The animal covers all four pool quadrants in a pattern with frequent direction changes.
(c) Scanning: Characterized by direction changes, but the pattern is limited to a couple of quadrants or a central area of the pool.
(d) Chaining: The swimming pattern is executed at a fixed distance from the wall but closer to the pool’s center than in the thigmotaxis strategy.
(e) Focal search: The search pattern is confined to the target quadrant, characterized by a dense concentration of overlapping loops and turns.
(f) Focal wrong: A focal search performed in an incorrect quadrant.
(g) Perseverance: The animal persists in searching in the target quadrant of the CUE stage after the platform has been moved to the PT position.
(h) Direct search: A straight swim toward the platform location.

#### 2.2.3. Variables Associated with Swimming Strategies

##### Percentage of Swimming Strategies

The video analysis approach facilitated the identification of multiple strategies within a single trial. Thus, the percentage of time spent on each was calculated as follows:

Search Strategy = Time spent in swimming strategy (s)/Escape Latency (s)

##### Cognitive Flexibility

In the present work, we propose a measurement of cognitive flexibility, which was calculated as the percentage of time spent until the first strategy used to find the platform changed.

Cognitive Flexibility = Time spent in first strategy (s)/Escape Latency (s)

##### Non-Search Strategy Episodes

The number of circling and floating episodes in each trial was recorded.

### 2.3. Statistical Analysis

All analyses were performed using R software (version 4.3.2). We used the afex package (version 1.3.0) to analyze classic parameters via a mixed model analysis of variance (Mixed ANOVA). Genotype and sex were treated as between-subject factors, while days, trials, and ages were considered within-subject factors.

An ordered beta regression model (glmmTMB package version 1.1.5) [13,49] was used to analyze proportion-dependent variables such as the proportion of swimming strategies and cognitive flexibility. A set of multiple models was generated from simplest to most complex to examine the effects of sex, genotype, trial, and age on the dependent variables. Animal ID was included as a random effect to account for repeated measures and individual variability. An AICc model selection (bbmle package version 1.0.25.1) was then applied to identify the top models. 

A generalized linear mixed model (GLMM) with a negative binomial distribution was conducted to determine whether the number of non-search strategies (namely, circling and floating episodes) was influenced by the trial, sex, genotype, or age of the animals.

The DHARMa package (version 0.4.6) was used to examine the model fit and residual diagnostics. 

Post hoc analyses with multiple comparisons were performed using the emmeans package (version 1.8.4.1). The *p*-value was then adjusted using the Tukey method for multiple comparisons.

Data were considered statistically significant when the *p*-value was less than 0.05. 

## 3. Results

### 3.1. Survival Analysis

During the follow-up period, there was a 37.28% mortality rate, with a loss of 22 animals, with no genotype or sex differences (Fisher exact test, *p* = 1.000) since about half of them were NTg mice (9 females, 3 males) and the other were 3xTg-AD mice (5 females, 5 males).

### 3.2. Classical MWM Test Analysis

#### 3.2.1. CUE Stage

**Figure 2:**
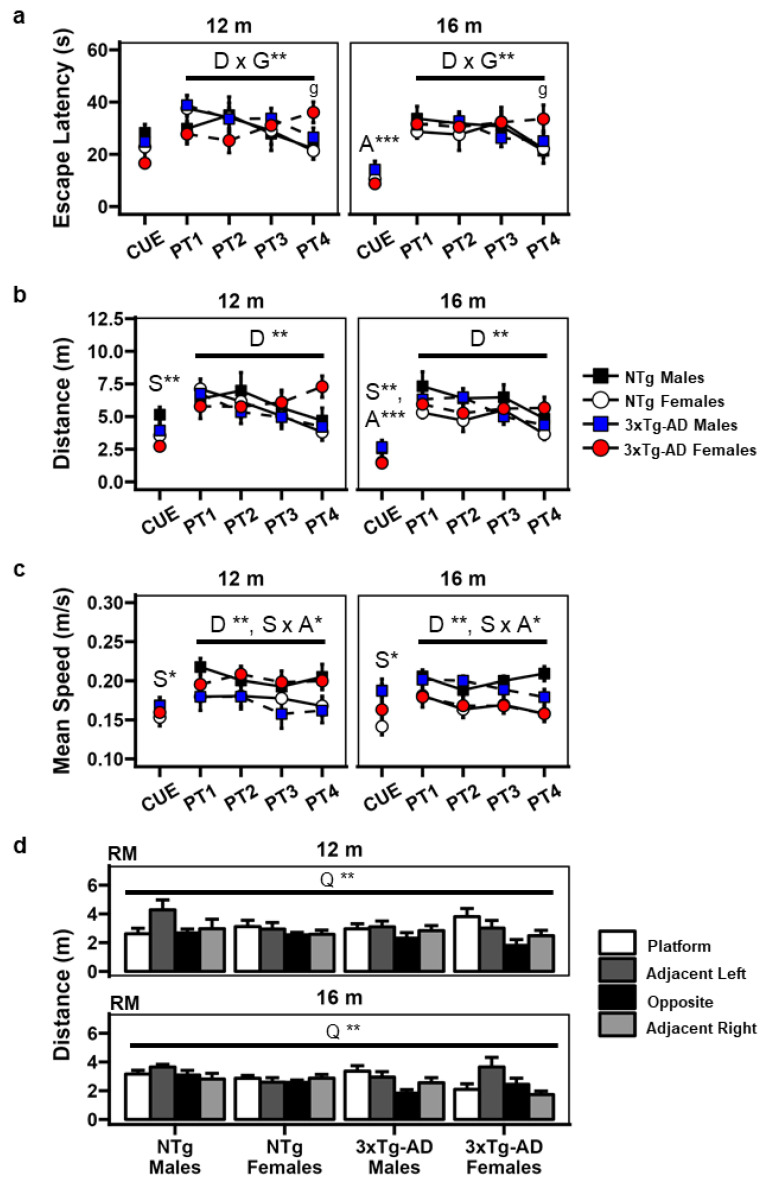
Classic parameters in the MWM test. (**a**) Escape latency. (**b**) Distance traveled. (**c**) Mean speed. (**d**) Distance traveled in the different quadrants during the removal stage. Mixed ANOVA: A, age effect; D, day effect; G, genotype effect; Q, quadrant effect; S, sex effect; g, genotype effect in stage PT4. * *p* < 0.05; ** *p* < 0.01; *** *p* < 0.001.

In the CUE stage, the mixed ANOVA test for escape latency revealed a significant age effect (Figure 2) [F(1,33) = 24.37, *p* < 0.001], indicating a decrease in escape latency for all animals (*n* = 37) at 16 months of age [16 m vs. 12 m: t(33) = −11.4, *p* < 0.001]. Upon analyzing the distance covered, significant sex [F(1,33) = 10.68, *p* = 0.003] and age effects [F(1,33) = 29.38, *p* < 0.001] were found (Figure 2); thus, females outperformed males [females vs. males: t(33) = −1.27, *p* = 0.003], and all animals decreased the distance swam with age [16 m vs. 12 m: t(33) = −1.79, *p* < 0.001]. 

Regarding mean speed, a significant sex effect was detected [F(1,33) = 5.24, *p* = 0.029], with females swimming slower than males [females v/s males: t(33) = −0.024, *p* = 0.029].

#### 3.2.2. PT Stage

A significant “stage” effect [F(2.87,94.73) = 4.23, *p* = 0.008] and a “stage × genotype” interaction effect [F(2.87,94.73) = 2.77, *p* = 0.48] were found in escape latency during the PT stage (Figure 2). Further analysis revealed that the “stage” effect accounted for differences between the PT1 and PT4 [t(33) = 6.38, *p* = 0.029] stages and the PT2 and PT4 [t(33) = 5.30, *p* = 0.031] stages in all animals (*n* = 37). The “stage × genotype” interaction can be explained by genotypical differences only on the fourth day of the task (PT4), where the 3xTg-Ad animals performed worse than the NTg animals [t(33) = 8.54, *p* = 0.022].

When the distance covered was analyzed, a significant stage effect [F(2.81,92.75) = 5.22, *p* = 0.003] indicated differences in the average values of the PT1 and PT4 [t(33) = 1.59, *p* = 0.011] stages and the PT2 and PT4 [t(33) = 1.07, *p* = 0.025] stages for all animals at both ages (*n* = 37).

In the mean swimming speed, a “stage” effect [F(1.94,63.87) = 5.46, *p* = 0.007] and a “sex × age” interaction effect [F(1,33) = 4.22, *p* = 0.048] were observed. The post hoc analysis revealed differences between the PT1 and PT3 [t(33) = 0.01, *p* = 0.018] stages and sex differences at 16 months of age only, with females swimming slower than males (t(33) = −0.03, *p* = 0.002). 

#### 3.2.3. RM Stage

Time and distance parameters were analyzed in each of the pool’s quadrants (Figure 2). A significant “quadrant” effect was observed for both time [F(2.18,72.03) = 4.08, *p* = 0.18] and distance [F(2.30,75.95) = 6.77, *p* = 0.001]. Further analysis revealed significant differences between the time spent in the adjacent left and opposite quadrants (AL vs. O: t(33) = −4.26, *p* = 0.002), the distance covered in the adjacent left and opposite quadrants (AL vs. O: t(33) = −4.547, *p* < 0.001), and the distance covered in the opposite and previous platform location quadrants (O vs. P: t(33) = −0.59, *p* = 0.025).

Additionally, a “sex × age” interaction effect was detected in the time spent [F(1,33) = 7.7, *p* = 0.009] within a zone surrounding the previous platform location by 1.5 cm. Consequently, females spent more time in this zone than males at 12 months of age (t(33) = 4.56, *p* = 0.014).

No differences were found in the mean swimming speed of the animals at any age.

### 3.3. Swimming Strategy Analysis

**Figure 3:**
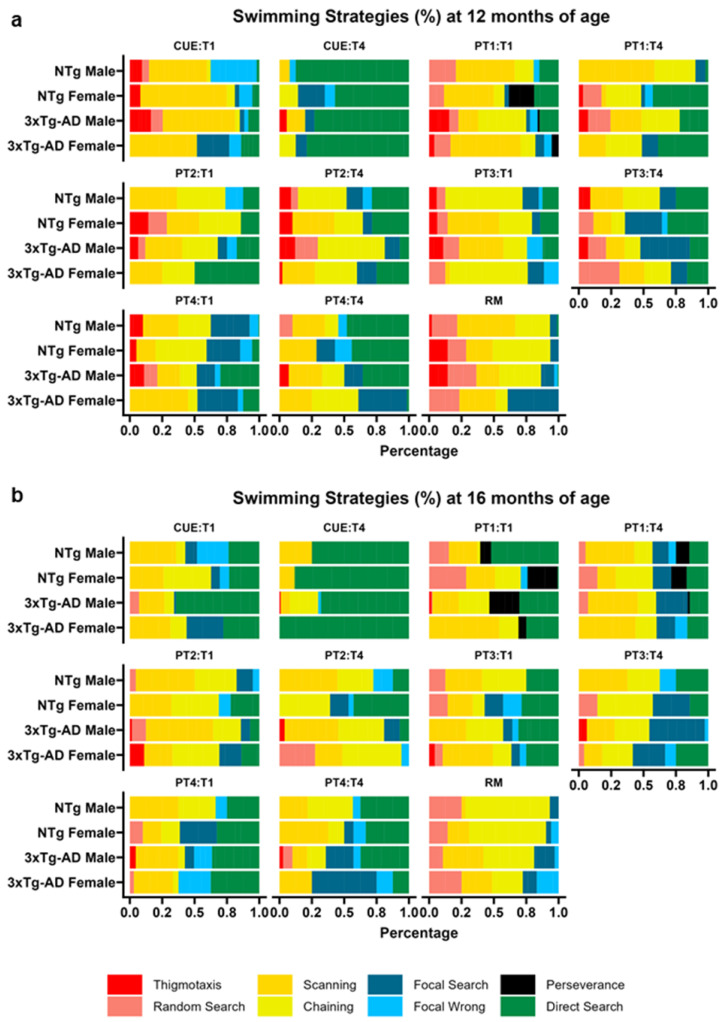
Comparative analysis of swimming strategies by group. Distribution of swimming strategies among different groups during the initial (T1) and final (T4) trials. Panels (**a**,**b**) represent the data collected at 12 and 16 months of age, respectively, showcasing the evolution in strategy adoption over time.

A total of 814 trials were analyzed during both MWM tests at 12 and 16 months of age. The distribution of strategies in each trial was analyzed (Figure 3). We found that the animals used up to four strategies per trial to find the platform (1.36 ± 0.56). A mixed-regression Poisson analysis showed a significant trial effect (*p* = 0.036), with fewer strategies used in T4 (1.25 ± 0.06) compared with T1 (1.43 ± 0.06), regardless of age, sex, genotype, or stage.

#### 3.3.1. Search Strategies

##### CUE Stage

The impact of sex, genotype, trials, and age on each strategy was evaluated. The optimal model varied depending on the strategy used as the dependent variable. Significant responses to predictors were observed in the random search, scanning, chaining, focal wrong, and direct search strategies (Table 1).

The genotype of the animals significantly influenced the proportion of random search, after adjusting for sex, age, and trials (*p* < 0.001). A pairwise test revealed that the 3xTg-AD mice were less likely to employ this strategy compared with their NTg counterparts (NTg vs. 3xTg-AD, odds ratio = 1.93, z = 18.646, *p* < 0.001).

An interaction effect between age and trial (*p* = 0.032) was detected in the scanning strategy. Specifically, a decrease in the likelihood of employing this strategy was observed in T4 compared with T1 at 12 months of age (odds ratio: 7.08, z = 3.73, *p* = 0.001).

An age effect (*p* = 0.002) was observed in the percentage of chaining, with an increase in its likelihood by 16 months of age (odds ratio: 0.55, z = −3.04, *p* = 0.002).

In the focal wrong strategy, a trial effect (*p* = 0.015) was found when adjusted by genotype, indicating that the likelihood of observing this strategy decreased by T4 compared with T1 (odds ratio = 2.70, z = 2.42, *p* = 0.015).

Regarding the direct search strategy, a model including age (*p* = 0.008) and trials (*p* < 0.001) was the top raked. Pairwise comparisons showed a significant increase in the likelihood of employing it by 16 months of age (odds ratio = 0.53, z = −2.65, *p* = 0.008) and by T4 (odds ratio = 0.23, z = −4.57, *p* < 0.001).

##### PT Stages

To evaluate the general changes in platform search patterns during this stage, data from PT1 to PT4 were pooled and analyzed, with a specific focus on T1 and T4. The response variables for each model included sex, genotype, trial, and age as predictors. Significant responses to some predictors were observed in the thigmotaxis, random search, and direct search strategies (Table 1).

A significant influence of age was found in the percentage of thigmotaxis used by the animals (*p* < 0.001). Specifically, a reduction was observed at 16 months of age compared with 12 months (odds ratio = 4.68, z = 4.40, *p* < 0.001). Additionally, age significantly influenced the percentage of random search (*p* = 0.008), with a reduction observed at 16 months (odds ratio = 1.73, z = 2.641, *p* = 0.008).

In the case of direct search, the top model included genotype and trial. A significant interaction effect was found (*p* = 0.002), with the NTg animals showing higher percentages than the 3xTg-AD animals in T4 (odds ratio: 1.81, z = 2.70, *p* = 0.035), regardless of sex and age.

##### RM Stage

During this stage, models that included sex, genotype, and age as predictors were evaluated. A significant influence of these predictors was found in the scanning, focal wrong, and direct search strategies (Table 1).

The age of the animals significantly influenced the percentage of scanning (*p* = 0.03), with a reduction observed at 16 months (odds ratio = 1.85, z = 2.16, *p* = 0.031).

For the focal wrong strategy, sex (*p* < 0.001), genotype (*p* < 0.001), and age (*p* < 0.001) significantly influenced this strategy. Specifically, an increase was observed at 16 months of age (odds ratio = 0.76, z = −43,924, *p* < 0.001), a higher percentage was observed in males (odds ratio = 1.42, z = 55,110, *p* < 0.001), and a higher percentage was observed in the non-transgenic (NTg) animals (odds ratio = 1.23, z = 33,454, *p* < 0.001).

Finally, the percentage of direct search was significantly influenced by genotype (*p* < 0.001), adjusted by age. Thus, the NTg animals showed a higher probability of using this strategy than the 3xTg-AD animals (odds ratio: 1.79, z = 4.451, *p* < 0.001).

#### 3.3.2. Non-Search Strategies

The effects of age, genotype, sex, and trial were examined on the response variables circling and floating in the different MWM stages (Table 2). 

##### Circling Behavior

Circling episodes were observed in 49.88% of the trials (406 out of 814), with an average duration of 3.23 s (SD: 6.37 s). A mixed-effect Poisson regression analysis during the CUE stage revealed significant influences from both trial (*p* < 0.001) and age (*p* < 0.001) on the number of circling episodes. Specifically, a decrease was noted by T4 (odds ratio = 4.67, z = 8.02, *p* < 0.001) and at 16 months of age (odds ratio = 2.26, z = 5.06, *p* < 0.001). In the PT stages, the number of circling episodes was significantly influenced by trial (*p* = 0.007), with a decrease observed in T4 compared with T1 (odds ratio = 1.44, z = 2.67, *p* = 0.007), and age (*p* = 0.013), with an increase noted at 16 months of age compared with 12 months (odds ratio = 0.70, z = −2.47, *p* = 0.013). During the RM stage, a significant interaction effect was found between genotype and age (*p* = 0.026). The post hoc analysis revealed a significant increase in the number of circling episodes only for the 3xTg-AD animals when comparing 12 and 16 months of age (odds ratio = 0.51, z = 0.24, *p* = 0.008)

##### Floating Behavior

Floating behavior was observed in 18.18% of the trials (148 out of 814), with an average duration of 1.92 s (SD: 6.28). During the CUE stage, the top model for the mixed-effect Poisson regression included only trial as a predictor, revealing a significant influence (*p* = 0.027). Specifically, a reduction was observed by T4 compared with T1 (odds ratio = 5.5, z = 2.28, *p* = 0.027). For the PT stages, the top model included both trial (*p* = 0.60) and age (*p* = 0.038) as predictors. The post hoc analysis showed a decrease in floating episodes by 16 months of age (odds ratio = 1.44, z = 2.07, *p* = 0.038). Finally, during the RM stage, no significant influences of sex, genotype, or age were found. 

#### 3.3.3. Cognitive Flexibility

Multiple mixed-model ordered beta regressions were built for each stage of the MWM test (Table 2). In the CUE stage, the top model included trial and sex as predictors, both of which showed a significant effect on the response variable (trial: *p* = 0.003, sex: *p* = 0.032). The post hoc analysis revealed an increase in the time taken to change the initial strategy across trials (Trial 1/Trial 4: odds ratio = 0.45, z= −2.91, *p* = 0.003) and that females required more time than males to change it (male/female: odds ratio = 0.64, z = −2.15, *p* = 0.032).

For the PT and RM stages, the null models, which included only the random effect of animal ID, were the top models. Thus, we assumed no influence of sex, genotype, age, or trials on cognitive flexibility during these stages.

## 4. Discussion

Our findings harnessed state-of-the-art statistical tools, including ordered beta regression, to sensitively detect sex and genotype cognitive nuances between normal and pathologically aged mice in a multi-strategy identification approach in different stages/paradigms of the MWM test. The present work also advocates for a comprehensive perspective that integrates classical variables—such as escape latency, distance covered, and swimming speed—with search and non-search strategies analysis to enhance our understanding of animal behavior under normal and pathological scenarios.

### 4.1. Classical MWM Test Analysis

In our analysis, quantitative variables gained significance primarily during the CUE stage, where the animals were introduced to the fundamental principles of the test. This phase is thus considered a period of training [50]. Importantly, females covered shorter distances than males before finding the platform, which may indicate a better habituation in this group. Furthermore, females exhibited slower swimming speeds than males.

Genotypical differences were detected when escape latency was analyzed during the last day of PT stages (PT4). Thus, 3xTg-AD mice required a longer time to find the platform at both 12 and 16 months of age. Nevertheless, we expected these differences in the acquisition curve to be more evident and detectable between both groups sooner, i.e., already at PT3 [51]. This absence of clear differences supports the inclusion of other variables for a more thorough interpretation of the test when aged animals are evaluated.

### 4.2. Relevant Swimming Strategies

Previous studies underscored the significance of categorizing swimming strategies during the MWM test in mouse models of AD [45,46]. Others were probably discouraged because of statistical limitations in handling the percentage of multi-strategic performances. Therefore, in most studies, the focus was primarily centered on the predominant strategy. In our previous investigations, we proposed an approach considering multiple strategies in each single trial [26]. In the present work, we further develop this approach and include statistical tools to deal with the challenges that such data can cause. This allowed us to identify genotypic differences at all stages of the test (CUE, PT, and RM). The main differences were observed during the PT stages, where NTg animals employed a direct search strategy more frequently than their 3xTg-AD counterparts. Such differences extend beyond traditional analysis, highlighting the importance of the use of statistical tools in a multi-strategy classification.

Although genotypic differences in random search and direct search were observed during the CUE and RM stages, respectively, the magnitude of these differences was minimal and thus not considered relevant.

Strategies associated with lower hippocampal involvement declined with age, while those more reliant on hippocampal function increased. These findings align with prior research [32,52,53], suggesting a transition in rodent swimming patterns from wall proximity (thigmotaxis) to proactive search strategies (direct search) following training. In fact, our laboratory has proposed that batteries of tests, mainly in longitudinal designs should be considered a “behavioral” cognitive training [54]. As further discussed below (Section 4.5), the current data support this understanding and provide a comprehensive analysis with further evidence on the functional aspects of this cognitive training.

### 4.3. Importance of Non-Search Strategies

Circling behavior was more prevalent than floating. As animals became accustomed to the test, the occurrence of both behaviors decreased, both across trials and upon retesting after a four-month interval. Notably, only the 3xTg-AD group exhibited an increase in circling behavior during the RM stage. This might be interpreted as an intensification of neuropsychiatric-like symptoms in the transgenic group when confronted with a non-escapable paradigm, potentially impacting the animals’ engagement in an active platform search.

Our observation of an association between circling episodes and 3xTg-AD animals aligns with previous research [27,48]. Despite this, in the 3xTg-AD animal set assessed in the present study, built on a C57BL/6 pure genetic background, we did not observe the persistent hyperactive phenotype often associated with this animal model when based in their original hybrid C57BL/6 × 129 background [26,42], as typically evidenced by a higher mean speed in the test. This would also explain other differences between this and the previous report with respect to the higher incidence of floating episodes previously reported in their NTg (C57BL/6 × 129) counterparts [48], which is not observed under this background strain. In this respect, variations in the stress response profiles among these animals because of their “survival bias” (see Section 4.6) could explain these differences in the floating behavior, a natural characteristic of mice swimming patterns that depends on basal anxiety levels and the behavioral profile [55,56].

### 4.4. Cognitive Flexibility

We noted sex variations in the transition between initial and subsequent swimming strategies during the CUE test. Specifically, females transitioned slower from their first strategy used to locate the platform compared with males. We propose that the CUE test challenges the cognitive flexibility of the animals. In the first experience, the test may be perceived as non-escapable, while learning opportunities make escape possible in subsequent trials. This observation suggests that males exhibit enhanced adaptability when navigating a novel environment, which could be a potential manifestation of cognitive flexibility.

While previous research has not specifically examined the transition of swimming strategies as a method for evaluating cognitive flexibility, the present work provides further evidence of the importance of assessing it in the test.

### 4.5. Retest Effect

Our investigation explored the role of prior experience in the test, specifically considering mouse strain characteristics. Thus, according to the classical and multi-strategy approach, animals seemed to improve their performance when retested. Previous longitudinal assessments involving Tg2576 [40] and APP/PS1 [41], which are AD-like models, consistently reveal stable performance upon retesting, similar to control groups. Zhang [57] underscores the protective effect of prior experience against non-cognitive decline in AD-like models. Notably, genotypic differences in 3xTg-AD mice manifest early but attenuate at advanced ages [43,54,58,59]. This reduction may stem from training effects, mortality bias, and age-related declines observed across both the transgenic and non-transgenic cohorts [41,60].

### 4.6. Limitations

Only animals that survived were included throughout the follow-up period. This “mortality” selection process skews our sample toward animals with distinct behavioral profiles, mainly regarding their stress response characteristics. As recently reported in our laboratory in 3xTg-AD and APP/swe mice, this “survival bias” renders a new window of observation in the experimental scenario [54,60,61].

Our methodology, although comprehensive, has certain limitations. There were two potential issues in the strategy identification i.e., it can be labor-intensive and may lack of reliability. However, we addressed this latter concern by ensuring inter-evaluator agreement. Moreover, it is important to highlight that current track analysis software may not be fully accurate for advanced feature analysis. As such, visual analysis continues to be a practical approach for intricate tasks.

### 4.7. Future Directions

One significant aspect for future research involves contrasting our findings by integrating the analysis of swimming strategies in other AD-like models within longitudinal studies. This approach will not only strengthen inter-study and inter-laboratory reliability but also offer a more detailed understanding of the observed behavioral patterns.

Previous reports from our laboratory [60] have depicted a neuropathological progression in 3xTg-AD mice of similar ages, with extracellular A-beta plaques present in 12-month-old females and 16-month-old males across various brain regions. Consequently, delving deeper into the neuroanatomical aspects contributing to the behavioral signatures observed in the MWM test can shed light on the mechanisms propelling these behaviors. Most importantly, as shown here, the longitudinal design that scrutinizes the effects of prior training in the test presents an intriguing avenue for research.

In agreement with our prior work on normal and AD-pathological aging [15,27,62,63], we consider that employing swimming strategies adds stronger methodological sensitivity to assess the effects of new compounds or interventions. It is essential to pay close attention to any alterations in the variables, such as those outlined here. This approach holds promise for the development of more effective preventive and/or therapeutic interventions for AD and normal aging.

## 5. Conclusions

We have successfully integrated GLMMs and ordered beta regression into the MWM test analysis to interpret the swimming patterns of the animals. This integration has allowed us to gain a more comprehensive understanding of mouse behavior in normal and AD-pathological aging. The results of our study underscore the effectiveness of this methodology. We believe that future research, particularly those focusing on the evaluation of normal and pathological aging in animals under intrinsic (i.e., sex-perspective) and extrinsic factors (i.e., social and environmental conditions, non-pharmacological and pharmacological interventions), will consider this approach as an indicator of the performance in the test.

## Figures and Tables

**Table 1:** Mixed-effects beta regression model coefficients for the top models describing the proportion of time spent on different swimming strategies. The significant influence of independent variables and pairwise comparisons is indicated in bold.

MWM Stage	Dependent Variable	Top Model	Independent Variables	Estimate	Predictor *p*-Value	Pairwise Comparison (Estimated Marginal Mean ± Standard Error)	*p*-Value Pairwise Comparison
CUE	Random search	~Sex + Genotype + Trial + Age + (1|animal ID)	Sex	−0.119	0.961		
			**Genotype**	−0.659	**<0.001**	**3xTg-AD (1.20 × 10^−6^ ± 0.0002) < NTg (2.32 × 10^−6^ ± 0.0004)**	**<0.001**
			Trial	−0.122	0.960		
			Age	−1.151	0.384		
	Scanning	~Trial + Age + Age: Trial + (1|animal ID)	**Trial**	−1.958	**<0.001**	**Trial 4 (0.27 ± 0.07) < Trial 1 (0.58 ± 0.04)**	**<0.001**
			Age	−0.579	0.028	16 m (0.43 ± 0.06) > 12 m (0.41 ± 0.07)	0.821
			**Age × Trial**	1.294	**0.031**	**12 m Trial 4 (0.21 ± 0.08) < 12 m Trial 1 (0.65 ± 0.04)**	**0.0011**
						16 m Trial 1 (0.51 ± 0.06) < 12 m Trial 1 (0.65 ± 0.04)	0.1267
						**16 m Trial 4 (0.35 ± 0.09) < 12 m Trial 1 (0.65 ± 0.04)**	**0.0078**
						**16 m Trial 1 (0.51 ± 0.06) > 12 m Trial 4 (0.21 ± 0.08)**	**0.0391**
						16 m Trial 4 (0.35 ± 0.09) > 12 m Trial 4 (0.21 ± 0.08)	0.5466
						16 m Trial 4 (0.35 ± 0.09) > 16 m Trial 1 (0.51 ± 0.06)	0.3127
	Chaining	~Age + (1|animal ID)	**Age**	0.601	**0.002**	**16 m (0.53 ± 0.02) > 12 m (0.39 ± 0.04)**	**0.002**
	Focal wrong	~Trial + Genotype + (1|animal ID)	**Trial**	−0.994	**0.015**	**Trial 4 (0.24 ± 0.10) < Trial 1 (0.46 ± 0.09)**	**0.015**
			Genotype	−0.857	0.062		
	Direct search	~Trial + Age + (1|animal ID)	**Trial**	1.414	**<0.001**	**Trial 4 (0.49 ± 0.07) > Trial 1 (0.19 ± 0.03)**	**<0.001**
			**Age**	0.638	**<0.001**	**16 m (0.40 ± 0.06) > 12 m (0.26 ± 0.04)**	**0.008**
PT	Thigmotaxis	~Age + (1|animal ID)	**Age**	−1.543	**<0.001**	**16 m (0.03 ± 0.02) < 12 m (0.13 ± 0.08)**	**<0.001**
	Random search	~Age + (1|animal ID)	**Age**	−0.546	**0.008**	**16 m (0.40 ± 0.05) < 12 m (0.54 ± 0.05)**	**0.008**
	Direct search	~Trial + Genotype + Trial: Genotype + (1|animal ID)	Trial	0.465	0.019	Trial 4 (0.21 ± 0.02) > Trial 1 (0.21 ± 0.02)	0.858
			Genotype	0.290	0.154		
			**Genotype × Trial**	−0.881	**0.002**	NTg Trial 4 (0.26 ± 0.03) > NTg Trial 1 (0.18 ± 0.03)	0.0894
						3xTg-AD Trial 1 (0.23 ± 0.02) > NTg Trial 1 (0.18 ± 0.03)	0.4844
						3xTg-AD Trial 4 (0.17 ± 0.03) < NTg Trial 1 (0.18 ± 0.03)	0.9437
						3xTg-AD Trial 1 (0.23 ± 0.02) < NTg Trial 4 (0.26 ± 0.03)	0.8111
						**3xTg-AD Trial 4 (0.17 ± 0.03) < NTg Trial 4 (0.26 ± 0.03)**	**0.0358**
						3xTg-AD Trial 4 (0.17 ± 0.03) < 3xTg-AD Trial 1 (0.23 ± 0.02)	0.1732
RM	Scanning	~Age + (1|animal ID)	**Age**	−0.615	**0.031**	**12 m (0.61 ± 0.05) > 16 m (0.46 ± 0.06)**	**0.031**
	Focal wrong	~Sex + Genotype + Age + (1|animal ID)	**Sex**	−0.350	**<0.001**	**Female (0.34 ± 1.01 × 10^−6^) < Male (0.43 ± 1.09 × 10^−6^)**	**<0.001**
			**Genotype**	−0.213	**<0.001**	**3xTg-AD (0.36 ± 7.37 × 10^−6^) < NTg (0.41 ± 1.32 × 10^−6^)**	**<0.001**
			**Age**	0.278	**<0.001**	**16 m (0.42 ± 7.6 × 10^−7^) > 12 m (0.35 ± 1.25 × 10^−6^** **)**	**<0.001**
	Direct	~Genotype + Age + (1|animal ID)	**Genotype**	−0.580	**<0.001**	**3xTg-AD (1.11 × 10^−8^ ± 0.02) < NTg (1.99 × 10^−8^ ± 0.03)**	**<0.001**
			Age	−0.310	1		

**Table 2:** Analysis of non-search strategies and cognitive flexibility. Significant influences of independent variables and results of pairwise comparisons are highlighted in bold.

Dependent Variable	MWM Stage	Top Model	Independent Variables	Estimate	Predictor *p*-Value	Pairwise Comparison (Estimated Marginal Mean ± Standard Error)	*p*-Value of Pairwise Comparison
Circling episodes	CUE	~ Trial + Age + (1|1|animal ID)	**Trial**	−1.54	**<0.001**	**T4 (0.67 ± 0.14) < T1 (3.12 ± 0.41)**	**<0.001**
			**Age**	−0.82	**<0.001**	**16 m (0.96 ± 0.17) < 12 m (2.17 ± 0.32)**	**<0.001**
	PT	~ Trial + Age + (1|animal ID)	**Trial**	−0.39	**<0.001**	**T4 (0.99 ± 0.13) < T1 (1.48 ± 0.18)**	**<0.001**
			Age	0.23	0.056		
	RM	~Sex + Genotype + Age + (Genotype × Age) + (1|animal ID)	Sex	0.125	0.713		
			Genotype	−0.743	0.171		
			Age	−0.007	0.986		
			**Genotype × age**	1.419	**0.026**	3xTg-AD 12 m (0.60 ± 0.27) < NTg 12 m (1.26 ± 0.50)	0.519
						NTg 16 m (1.25 ± 0.53) < NTg 12 m (1.26 ± 0.50)	1
						3xTg-AD 16 m (2.46 ± 0.71) > NTg 12 m (1.26 ± 0.50)	0.402
						3xTg-AD 12 m (0.60 ± 0.27) < NTg 16 m (1.25 ± 0.53)	0.524
						**3xTg-AD 12 m (0.60 ± 0.27) < 3xTg-AD 16 m (2.46 ± 0.71)**	**0.008**
						3xTg-AD 16 m (2.46 ± 0.71) > NTg 16 m (1.25 ± 0.53)	0.383
Floating episodes	CUE	~Trial + (1|animal ID)	**Trial**	−1.704	**0.027**	**T4 (0.02 ± 0.02) < T1 (0.11 ± 0.03)**	**0.027**
	PT	~Trial + Age + (1|animal ID)	Trial	0.091	0.602		
			**Age**	−0.368	**0.038**	**16 m (0.075 ± 0.02) < 12 m (0.11 ± 0.04)**	**0.038**
	RM	~1 + (1|animal ID)					
Cognitive flexibility	CUE	~Trial + Sex + (1|animal ID)	**Trial**	0.803	**0.004**	**T4 (0.72 ± 0.05) > T1 (0.53 ± 0.03)**	**0.004**
			**Sex**	0.441	**0.032**	**Female (0.68 ± 0.04) < Male (0.58 ± 0.04)**	**0.032**
	PT	~1 + (1|animal ID)					
	RM	~1 + (1|animal ID)					

## Data Availability

Data are contained within the article.
